# Simvastatin attenuates tibial bone loss in rats with type 1 diabetes and periodontitis

**DOI:** 10.1186/s12967-018-1681-6

**Published:** 2018-11-09

**Authors:** Ae Ri Kim, Ji-Hye Kim, Aeryun Kim, Yongsung Sohn, Jeong-Heon Cha, Eun-Jung Bak, Yun-Jung Yoo

**Affiliations:** 1 Department of Oral Biology, Yonsei University College of Dentistry, 134 Sinchon dong, Seodaemun-gu, Seoul, 120-752 Republic of Korea; 2 Department of Applied Life Science, The Graduate School, Yonsei University, Seoul, Republic of Korea; 3 BK21 PLUS Project, Yonsei University College of Dentistry, Seoul, Republic of Korea; 4 Department of Dental Hygiene, Jeonju Kijeon College, Jeonju, Republic of Korea; 5 DONG-A Pharm, Yongin-si, Gyeonggi-do Republic of Korea; 6 Microbiology and Molecular Biology, Key Laboratory of Oral Medicine, Guangzhou Institute of Oral Disease, Stomatology Hospital of Guangzhou Medical University, Guangzhou, China

**Keywords:** Diabetes, Periodontitis, Bone loss, Sclerostin, Simvastatin

## Abstract

**Background:**

Diabetes induces long bone loss and aggravation of periodontitis-induced alveolar bone loss. Simvastatin (SIM), which is a lipid-lowering agent is known to have an anabolic effect on bone. Therefore, we investigated effect of SIM on tibial and alveolar bone loss in type 1 diabetic rats with periodontitis.

**Methods:**

Rats were divided into control (C), diabetes with periodontitis (DP), and diabetes with periodontitis treated with SIM (DPS) groups. DP and DPS groups were intravenously injected with streptozotocin (50 mg/kg), and C group was injected with citrate buffer. Seven days later (day 0), periodontitis was induced by ligatures of mandibular first molars. DP and DPS groups were orally administered vehicle or SIM (30 mg/kg) from day 0 to days 3, 10, or 20. Alveolar and tibial bone loss was measured using histological and m-CT analysis alone or in combination. Osteoclast number and sclerostin-positive osteocytes in tibiae were evaluated by tartrate-resistant acid phosphatase and immunohistochemical staining, respectively. Glucose, triglyceride (TG), cholesterol (CHO), and low-density lipoprotein (LDL) were evaluated.

**Results:**

Consistent with diabetes induction, the DP group showed higher glucose and TG levels at all timepoints and higher CHO levels on day 20 than C group. Compared to the DP group, the DPS group exhibited reduced levels of glucose (day 3), TG (days 10 and 20), CHO, and LDL levels (day 20). Bone loss analysis revealed that the DP group had lower bone volume fraction, bone mineral density, bone surface density, and trabecular number in tibiae than C group at all timepoints. Interestingly, the DPS group exhibited elevation of these indices at early stages compared to the DP group. The DPS group showed reduction of osteoclasts (day 3) and sclerostin-positive osteocytes (days 3 and 20) compared with the DP group. There was no difference in alveolar bone loss between DP and DPS groups.

**Conclusions:**

These results suggest that SIM attenuates tibial, but not alveolar bone loss in type 1 diabetic rats with periodontitis. Moreover, attenuation of tibial bone loss by SIM may be related to inhibition of osteoclast formation and reduction of sclerostin expression.

## Background

Simvastatin (SIM) inhibits 3-hydroxy-3-methylglutaryl coenzyme A reductase and subsequently decreases cholesterol synthesis [1]. Thus, SIM is prescribed as an anti-hyperlipidemic agent for reducing cardiovascular disease. In addition, SIM has anti-inflammatory, angiogenic, and bone anabolic actions, which have generated interest in clinical fields related to inflammatory, ischemic, and bone diseases [2–4].

Periodontitis is an inflammatory disease that induces alveolar bone loss. In periodontitis animal models, SIM treatment has a beneficial effect on alveolar bone level [5–8]. Additionally, SIM treatment resulted in improved radiographic defect depth in periodontitis patients [1, 9, 10]. The prevalence or severity of periodontitis is affected by systemic conditions including menopause, medication, smoking, and metabolic syndrome. SIM treatment prevents alveolar bone loss in ovariectomized rats with periodontitis [11] and recovers cyclosporine A-induced alveolar bone loss in periodontitis rats [12]. In smokers with chronic periodontitis, SIM treatment in combination with nonsurgical treatment improves bone fills [13]. In addition, SIM treatment inhibits lipopolysaccharide (LPS)-induced alveolar bone loss in Zucker fatty rats with increased body weight and high cholesterol level [14].

A complication of diabetes mellitus is bone fractures [15]. As an anabolic agent, SIM is effective in the treatment of facture healing [16]. However, the activity of SIM on bone anabolism under diabetic conditions remains controversial. Bone mineral density (BMD) in SIM-treated subjects with type 2 diabetes mellitus did not show a significant difference compared with untreated subjects with type 2 diabetes mellitus [17]. In contrast, SIM application on calvaria defects was shown to enhance bone defect healing in rats with streptozotocin (STZ)-induced diabetes [18].

Periodontitis is another complication of diabetes mellitus. Diabetic patients with difficulty controlling glucose levels are more likely to suffer from periodontitis compared with those with sufficient diabetes control and healthy subjects [19, 20]. One group reported that local SIM application improved alveolar bone fill in patients with type 2 diabetes and chronic periodontitis when combined with scaling and root planing [21]. Until now, systemic SIM administration in diabetic subject with periodontitis has not been evaluated. As diabetic patients can show hyperlipidemia, aggravated alveolar bone loss due to periodontitis, and long bone loss, it is necessary to evaluate the effect of systemic administration of SIM. Studying the effect of systemic SIM administration on bone loss using a diabetic animal model with periodontitis may have translational utility in the clinical field. Animal models of STZ-induced diabetes display pancreatic β-cell destruction and are commonly used as an experimental approach to the study of human type 1 diabetes; however, extrapolation of findings from these models to humans remains challenging due to potential toxicity in the liver and kidneys [22]. Nevertheless, rats with STZ-induced type 1 diabetes and periodontitis provide a good model for observing coincident tibial and alveolar bone loss. In our previous study, rats with STZ-induced diabetes and periodontitis showed increased alveolar bone loss that was attributed to elevation of bone resorption and inhibition of bone formation after the induction of periodontitis compared with control rats [23]. Based on these observations, we hypothesized that SIM could attenuate bone loss during the progression of type 1 diabetes and periodontitis and determined the effect of systemic SIM treatment on alveolar and tibial bone loss using rats with STZ-induced type 1 diabetes and ligature-induced periodontitis.

## Methods

### Animal model with diabetes and periodontitis and simvastatin treatment

**Fig. 1:**
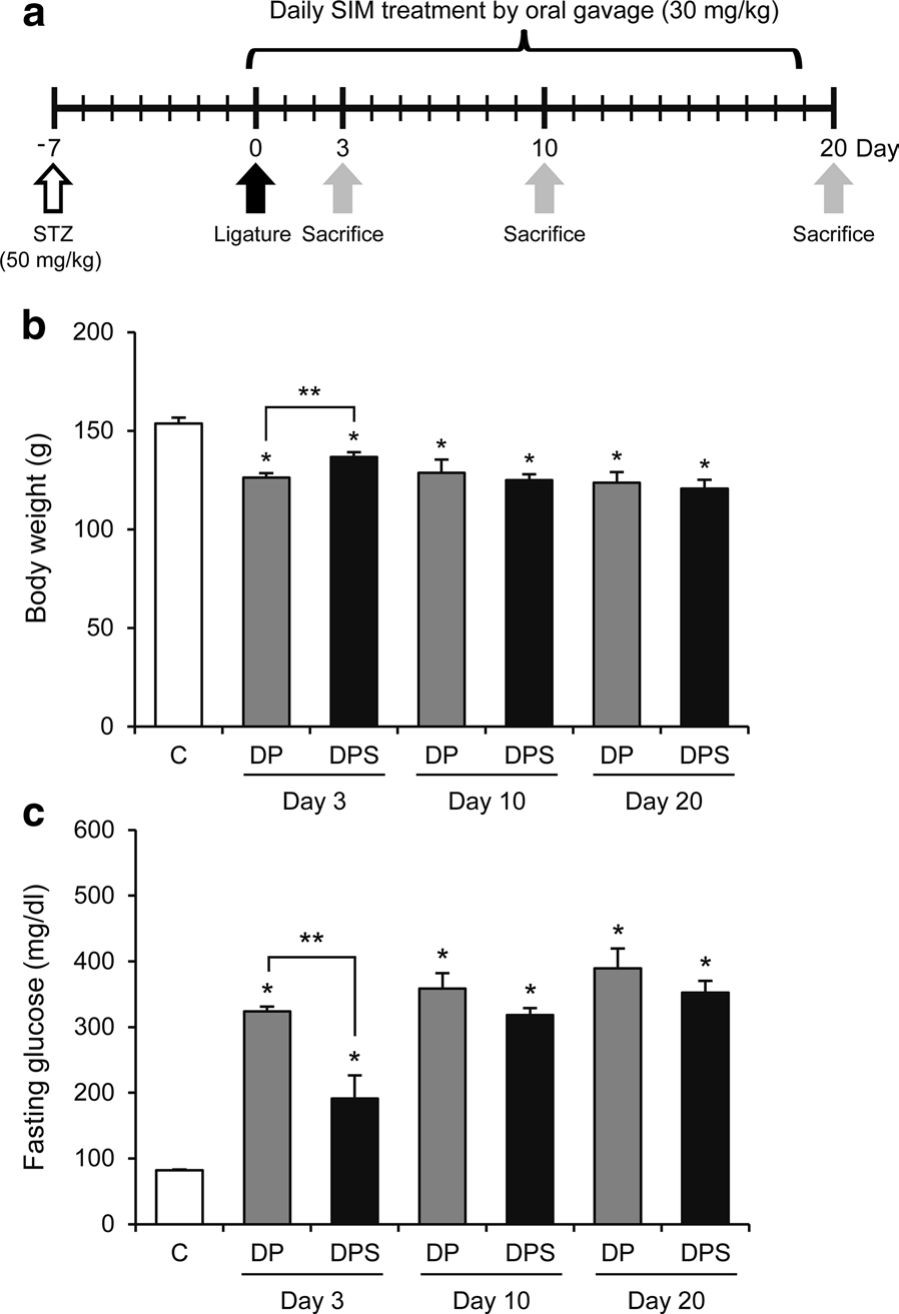
Experimental scheme and the effects of SIM on body weight and fasting glucose level. Rats were divided into three groups: C, DP, and DPS. **a** Experimental scheme: 7 days after induction of diabetes by STZ (50 mg/kg) injection, periodontitis was induced by ligatures of the mandibular first molars using dental floss (day 0). After placement of the ligatures, the DPS group was orally administered SIM (30 mg/kg) daily for a duration of 3, 10, or 20 days. **b** Body weight and **c** fasting glucose level in each group. Data are presented as the mean ± SE. *P < 0.05 compared with the C group. **P < 0.05

F344 rats (male, 6 weeks old) were purchased from Shizuoka Laboratory Center (Shizuoka, Japan). To minimize distress, all rats were housed three per a cage in specific pathogen-free conditions on a 12 h light/dark cycle at constant temperature (22 °C) and received standard chow and water ad libitum. After acclimation for 1 week, rats were randomly assigned to three groups: control group (C), diabetes with periodontitis group (DP), or diabetes with periodontitis treated with SIM group (DPS). After fasting for 18 h, DP and DPS groups were intravenously injected with STZ (50 mg/kg; Sigma-Aldrich, St. Louis, MO, USA) dissolved in 0.1 M citrate buffer. The C group was injected with citrate buffer alone. Seven days after injection (day 0), blood glucose levels were measured from the tail vein using a glucometer (Accu-Check active system, Roche, Mannheim, Germany). Rats with fasting blood glucose levels over 300 mg/dl were considered diabetic. On day 0, all rats were intraperitoneally injected with a mixture of zoletil 50 (30 mg/kg; Virbac, Carros, France) and rompun (10 mg/kg; Bayer Korea, Ansan, Korea). Following injection, periodontitis was induced in the DP and DPS groups by ligatures of the mandibular first molars with dental floss to induce biofilm formation. SIM (30 mg/kg/day, Chong Kun Dang Pharm, Seoul, Korea) was given daily via oral gavage to the DPS group, and saline was given to the DP group. The C group was sacrificed on day 0 (n = 6), and DP and DPS groups were sacrificed on days 3 (DP, n = 6; DPS, n = 7), 10 (DP, n = 4; DPS, n = 4), and 20 (DP, n = 7; DPS, n = 9). The experimental protocol is presented in Fig. 1a. The maintenance of diabetes was confirmed by measuring body weight and the fasting glucose level during the experimental period. The animal protocols were approved by the Institutional Animal Care and Use Committee of Yonsei University (Approval Number: 2015-0345).

### Serum biochemistry

Serum was obtained from all rats under fasting conditions at sacrifice. Levels of serum triglyceride (TG), total cholesterol (CHO), and low-density lipoprotein (LDL) were measured using a Konelab prime 30i automated analyzer (Thermo scientific, Waltham, MA, USA).

### Histological analysis of alveolar and tibial bones

Mandibles dissected from rats were fixed with 10% neutral-buffered formalin for 24 h and embedded in paraffin after decalcification with 10% ethylenediaminetetraacetic acid (EDTA) for 2 months. Sagittal sectioned mandibles were cut at 4 μm thickness. Sections, including clear dental pulp of the mesial and distal roots of the first molar, were selected and stained with hematoxylin and eosin (H&E). For determination of alveolar bone loss, the distance between the cementoenamel junction (CEJ) and the alveolar bone crest (ABC) was measured in the distal area, and the alveolar bone area was measured in the region of interest (ROI) of the furcation. Then, the alveolar bone area in the ROI was divided by the ROI. The ROI was 0.8 mm downward from the top of the furcation. For the evaluation of alveolar bone formation, the light pink unmineralized osteoid area between the alveolar bone surface and osteoblasts was measured in the ROI, which was 0.5 mm downward from the ABC in the furcation. For the determination of tibial bone loss, tibiae were fixed with 10% neutral-buffered formalin. After 24 h of fixation, left tibiae were decalcified with 10% EDTA for 1 month after which paraffin embedding was performed. The paraffin blocks were sectioned as previously described, and the sections containing the clear proximal epiphyseal growth plate were selected and H&E stained. The ROI for trabecular bone extended 1.0 mm from 1.0 mm below the growth plate toward the diaphysis (excluding the outer cortical shell). The area of remaining trabecular bone in the ROI was measured and then divided by the ROI. An Aperio AT2 Digital Whole Slide scanner (Leica Microsystems Inc., Buffalo Grove, IL, USA) and Aperio ImageScope software (version 12.3.2.2013, Aperio Technologies Inc., Vista, CA, USA) were used for the slide scanning and analysis, respectively.

### Micro-computed tomography analysis of tibiae

Right tibiae were scanned using micro-computed tomography (m-CT, Skyscan1076, Kontich, Belgium). The scanning parameters were set at 70 kV and 139 μA with an exposure time of 1475 ms and a resolution of 18 μm. The trabecular bone structures extending 2 mm from 0.7 mm below the epiphyseal growth plate were chosen as the ROI [24], and three-dimensional images of the specimens were reconstructed. Bone volume fraction (BVF), BMD, bone surface density (BSD), and trabecular number (Tb. N) in the ROI were calculated using the NRecon software (Bruker SkyScan, Aartselaar, Belgium).

### Tartrate-resistant acid phosphatase staining

To identify osteoclast formation in tibiae sections, tartrate-resistant acid phosphatase (TRAP) staining was performed using a TRAP stain kit (Wako chemicals, Osaka, Japan). Methyl green (Trevigen, Gaithersburg, MD, USA) was used for the counterstaining, and slides were scanned as described above. The number of TRAP-positive cells in the ROI were counted along the trabecular bone surface, divided by the bone length, and then divided by the percentage of bone area remaining in the ROI. The ROI was area extended 1.0 mm from 1.0 mm below the growth plate toward the diaphysis (excluding the outer cortical shell).

### Immunohistochemistry staining

To assess sclerostin expression in tibiae, tibiae sections were deparaffinized in xylene and rehydrated through ethanol series. Sections were incubated with Trypsin Enzymatic Antigen Retrieval Solution (Abcam, Cambridge, UK) for 15 min at 37 °C followed by immersion in 3% H_2_O_2_ in methanol for 20 min to block endogenous peroxidase. After blocking in normal horse serum (Vector Laboratories, Burlingame, CA, USA) for 20 min, the sections were incubated overnight at 4 °C with goat anti-sclerostin antibody (1:50 dilution, R&D Systems, Minneapolis, MN, USA). After incubation with anti-goat secondary antibody (Vector Laboratories) for 20 min, sections were developed with 3,3-diaminobenzidine tetrahydrochloride substrate chromogen system (DAKO, Botany, Australia) and counterstained with methyl green. Slides were scanned as described above. The number of sclerostin-positive osteocytes in the ROI was divided by the number of total osteocytes in the ROI. The ROI was the same as the ROI for osteoclast counting.

### Statistical analysis

All statistical comparisons were made using a statistical analysis program (SPSS, Chicago, IL, USA). Significant differences were determined using one-way analysis of variance with Scheffé’s post hoc test. A value of *P* < 0.05 was considered statistically significant. Data are expressed as the mean ± standard error (SE).

## Results

### SIM reduces body weight and fasting glucose level at an early stage in diabetic rats with periodontitis

Body weight was reduced and fasting glucose level was elevated in the DP and DPS groups compared with the C group for the duration of the experimental period (Fig. 1b, c). Interestingly, the DPS group had higher body weight and lower fasting glucose level than the DP group on day 3.

### Serum TG, CHO, and LDL levels are reduced with SIM treatment

**Fig. 2:**
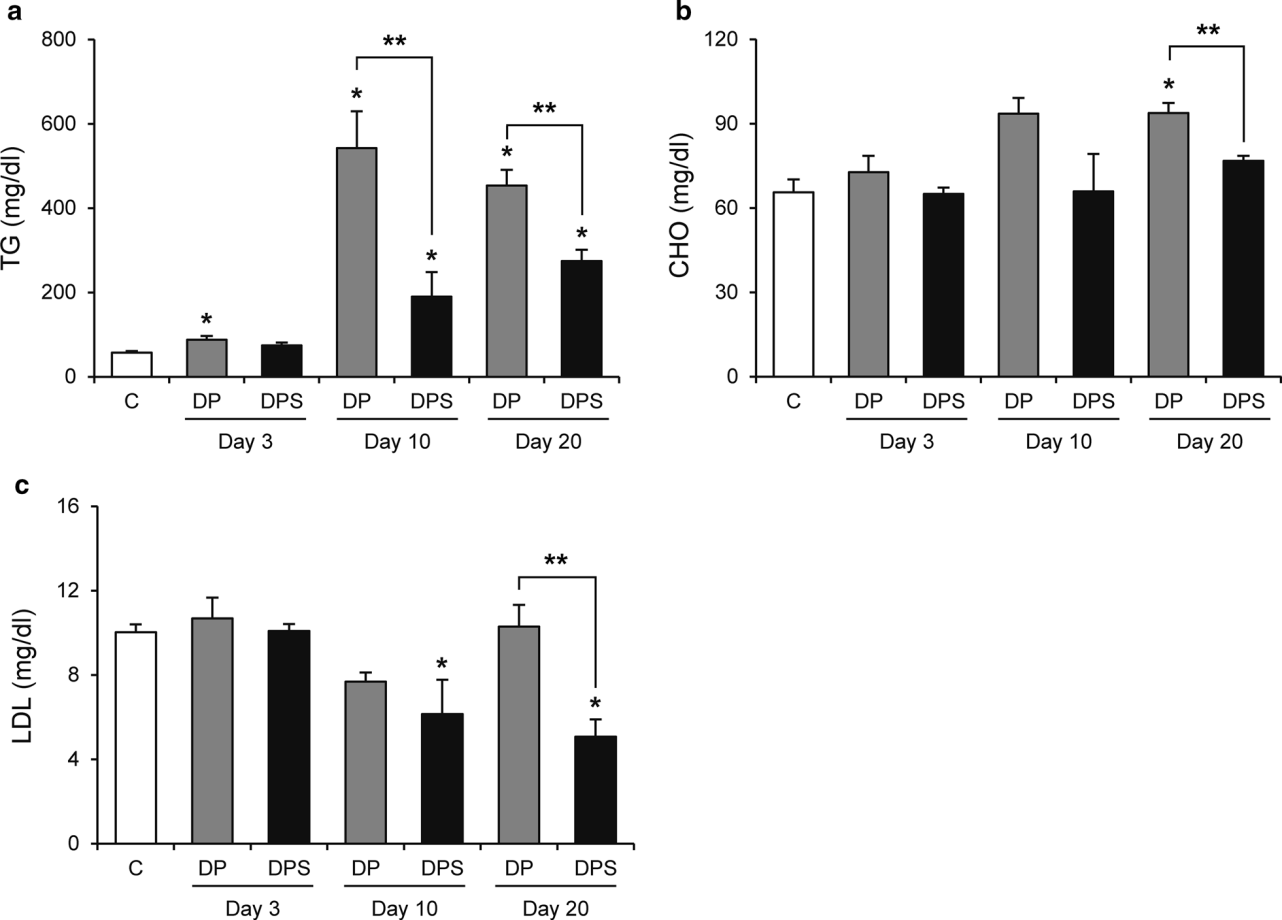
The effects of SIM on serum lipid levels. The serum levels of **a** TG, **b** CHO, and **c** LDL of each group. Data are presented as the mean ± SE. *P < 0.05 compared with the C group. **P < 0.05. (C, n = 6; day 3: DP, n = 6 and DPS, n = 7; day 10: DP, n = 4 and DPS, n = 4; day 20: DP, n = 7 and DPS, n = 9)

TG level was higher in the DP group than the C group during the experimental period but was lower in the DPS group than the DP group on days 10 and 20 (Fig. 2a). On day 20, CHO level was higher in the DP group than the C group, but was lower in the DPS group than the DP group (Fig. 2b). In terms of LDL, the DPS group also showed lower level than the DP group on day 20 (Fig. 2c).

### Alveolar bone loss is not attenuated by SIM treatment

**Fig. 3:**
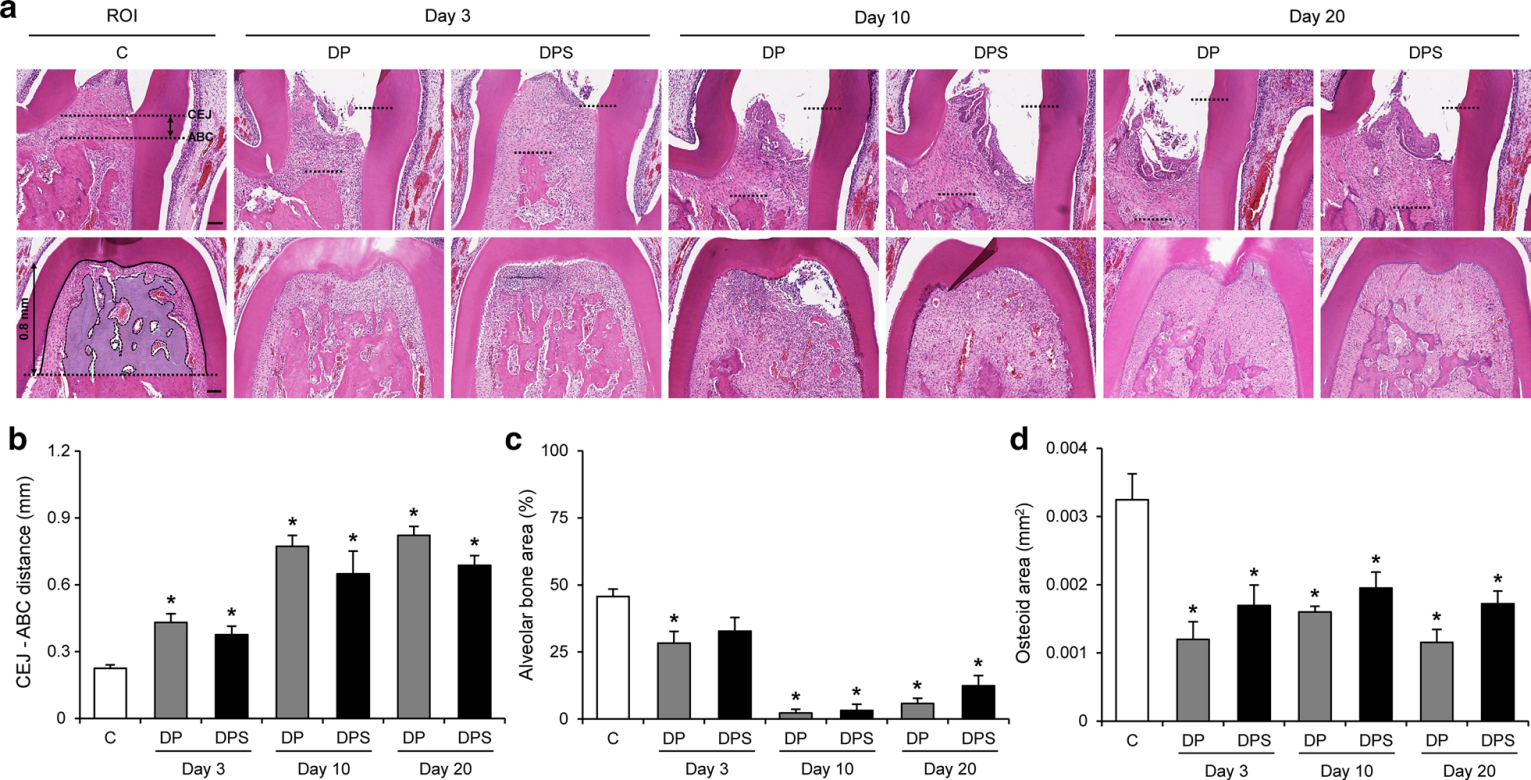
The effects of SIM on alveolar bone loss and osteoid area. **a** ROI and representative images for the measurement of alveolar bone loss (H&E staining). The CEJ-ABC distance in the distal area (upper panels, black arrow) and the alveolar bone area (pale blue color) in the ROI (black line) of the furcation (lower panels) were measured in each rat. **b** The measurement of the CEJ-ABC distance in the distal area of each group. **c** Alveolar bone area as a percent of the ROI in the furcation of each group. **d** Osteoid area in the furcation of each group. Data are presented as the mean ± SE. Scale bar = 100 μm. *P < 0.05 compared with the C group. (C, n = 6; day 3: DP, n = 6 and DPS, n = 7; day 10: DP, n = 4 and DPS, n = 4; day 20: DP, n = 7 and DPS, n = 9)

The effect of SIM on alveolar bone loss and formation of first molars was estimated by measuring the CEJ- ABC distance in the distal area (Fig. 3a, b), the alveolar bone area in the furcation (Fig. 3a, c), and the osteoid area in the furcation (Fig. 3d). The CEJ-ABC distance was higher in the DP and DPS groups than the C group, and there was no difference between the DP and DPS groups. Alveolar bone area in the DP group was lower than the C group for the duration of the experimental period, and the DPS group was lower on days 10 and 20. However, there was no difference between the DP and DPS groups. Similarly, the osteoid area was lower in the DP and DPS groups than the C group for the entire experimental period, and there was no difference between the DP and DPS groups.

### SIM reduces tibial bone loss

**Fig. 4:**
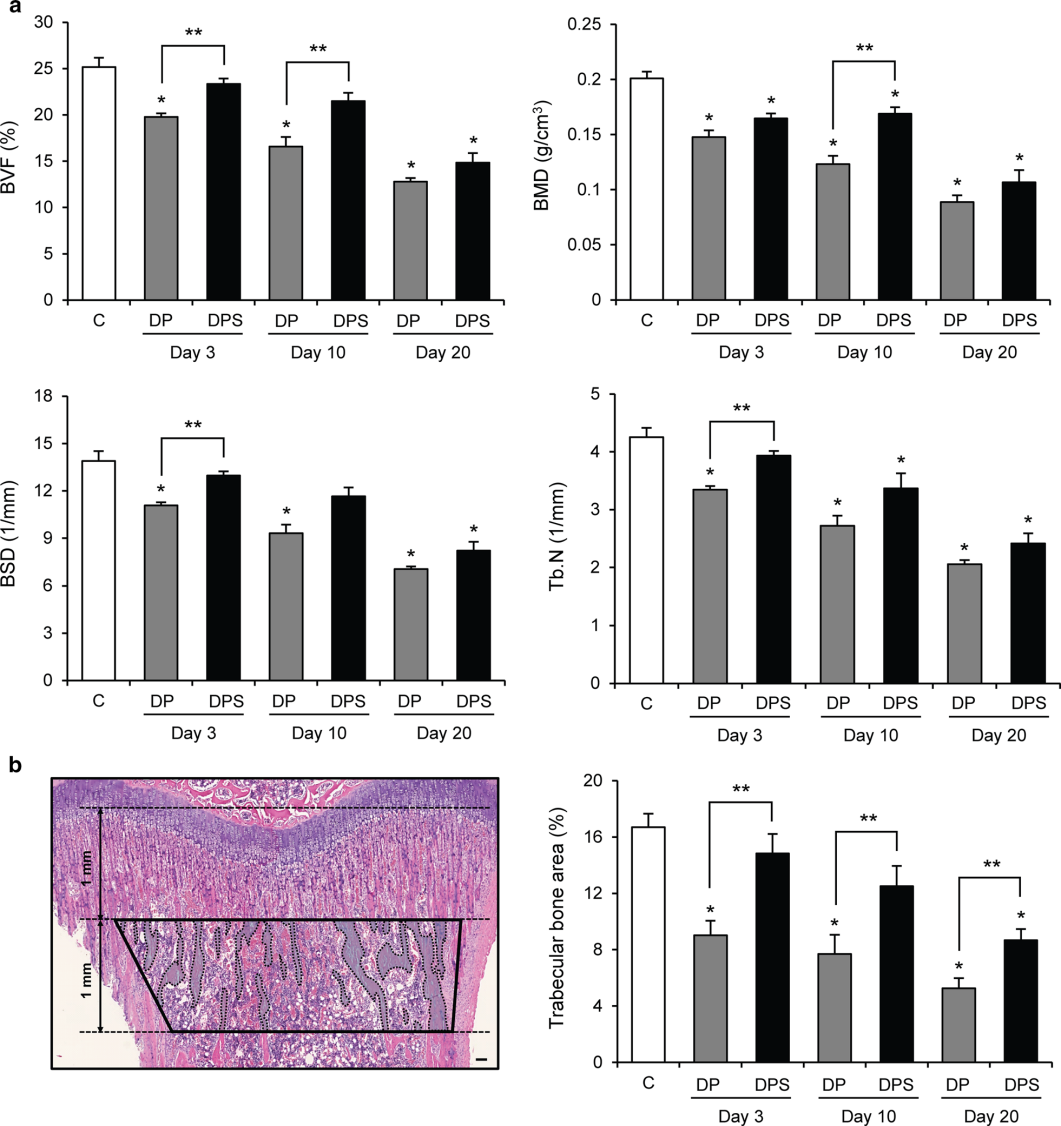
The effects of SIM on tibial bone loss. **a** Parameters of m-CT analysis: BVF, BMD, BSD, and Tb. N in each group. (C, n = 5; day 3: DP, n = 5 and DPS, n = 5; day 10: DP, n = 4 and DPS, n = 4; day 20: DP, n = 5 and DPS, n = 6). **b** Trabecular bone area (pale blue color) in the ROI (black line) of tibiae (left panel, H&E staining, scale bar = 100 μm) and measurement of trabecular bone area in each group (right panel; C, n = 6; day 3: DP, n = 6 and DPS, n = 7; day 10: DP, n = 4 and DPS, n = 4; day 20: DP, n = 7 and DPS, n = 9). Data are presented as the mean ± SE. *P < 0.05 compared with the C group, **P < 0.05

The effect of SIM on tibial bone loss was estimated by m-CT (Fig. 4a) and histological analysis (Fig. 4b). In m-CT analysis, levels of BVF, BMD, BSD, and Tb. N were lower in the DP group than the C group during the experimental period. On day 3, the DPS group showed higher levels of BVF, BSD, and Tb. N than the DP group. Additionally, on day 10, the DPS group showed increased levels of BVF and BMD compared with the DP group. Finally, on day 20, there was no difference between the DPS and DP groups. In histological analysis, tibial bone area in the DP group was lower than that of the C group at all timepoints, but the DPS group was only lower than the C group on day 20. Interestingly, tibial bone area was higher in the DPS group than the DP group for the duration of the experimental period.

### Osteoclast formation and sclerostin expression in tibiae are reduced with SIM treatment

**Fig. 5:**
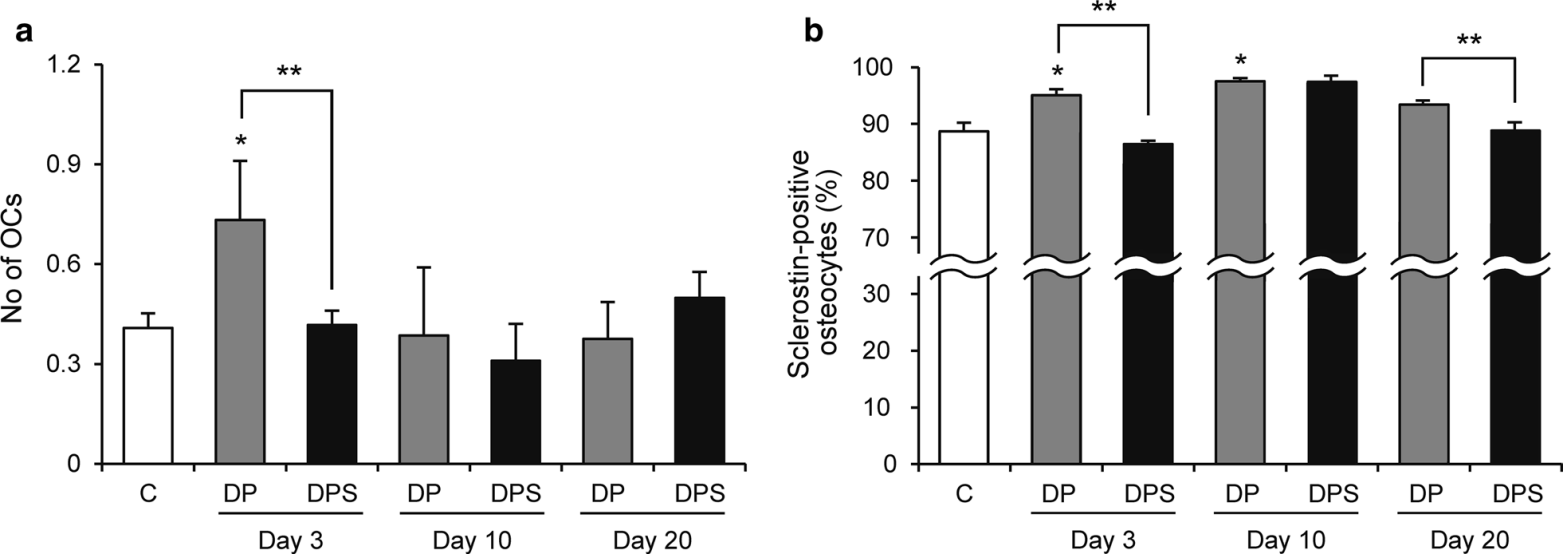
The effects of SIM on osteoclast formation and sclerostin expression in tibiae. **a** The number of TRAP-positive osteoclasts and **b** percentage of sclerostin-positive osteocytes in each group. Data are presented as the mean ± SE. *P < 0.05 compared with the C group, **P < 0.05. *No* number; *OCs* osteoclasts. (C, n = 6; day 3: DP, n = 6 and DPS, n = 7; day 10: DP, n = 4 and DPS, n = 4; day 20: DP, n = 7 and DPS, n = 9)

The effect of SIM on osteoclast formation in tibiae was estimated by TRAP staining (Fig. 5a). On day 3, the number of TRAP-positive osteoclasts was higher in the DP group than the C group and was lower in the DPS group than the DP group. There was no difference among the three groups on days 10 and 20. Sclerostin expression in osteocytes was estimated by immunohistochemistry and subsequent quantification of positive cells relative to the total number of osteocytes (Fig. 5b). On days 3 and 10, the proportion of sclerostin-positive osteocytes was higher in the DP group than the C group. Further, sclerostin-positive osteocyte proportions were reduced in the DPS group compared with the DP group on days 3 and 20.

## Discussion

Patients with poorly controlled type 1 diabetes often present with elevated TG, CHO, and LDL levels [25]. Mice with STZ-induced diabetes also show higher levels of glucose, TG, CHO, and LDL compared with normal mice [26]. Consistent with these reports, our study found that STZ-treated rats with periodontitis exhibited high levels of glucose, TG, and CHO as well as severe weight loss, indicating successful induction of diabetes in our model. In the previous study, the lipid-lowering agent, SIM, reduced CHO and LDL in patients with type 1 diabetes [25]. In this study, systemic SIM treatment attenuated weight loss and decreased glucose, TG, CHO, and LDL levels in STZ-treated rats with periodontitis. The beneficial effects of SIM on weight loss and high glucose level appeared transiently at an early stage. However, the anti-hyperlipidemic effects of SIM were more prominent for the duration of administration. These results suggest that systemic SIM treatment had promising effects during the experimental period in type 1 diabetic rats with periodontitis.

Bone loss is determined by bone resorption of osteoclasts and bone formation of osteoblast. Increases in cathepsin K and the ratio of receptor activator of nuclear factor kappa-Β ligand (RANKL) to osteoprotegerin in tibiae were noted in mice with STZ-induced diabetes [24]. Another group also reported that mice with STZ-induced diabetes had elevated expression of osteoclast-specific genes (matrix metallopeptidase-9, carbonic anhydrase II, RANKL) in tibiae than non-diabetic mice [27]. These studies suggest that bone resorption in tibiae increases under type 1 diabetic conditions. In accordance with these reports, we found that the DP group displayed reduced tibial bone volume and area at all timepoints compared with the C group. The DPS group showed higher BVF, BSD, Tb. N, and BMD levels on days 3 and 10 as well as greater tibial bone area on days 3, 10, and 20 compared with the DP group. The number of TRAP-positive osteoclasts in tibiae was higher in the DP group than in the C group and was lower in the DPS groups than in the DP group on day 3. These results suggest that systemic SIM treatment attenuated tibial bone loss and osteoclast formation under type 1 diabetic conditions at the early stage and the effect of SIM may weaken at later stage with severe bone loss.

Osteoblasts play an important role in bone formation through producing bone matrix proteins. In previous studies, SIM treatment improved bone formation by inhibiting osteoblast apoptosis through the transforming growth factor (TGF)/SMAD family member 3 signaling pathway as well as by stimulating osteoblast differentiation through induction of bone morphogenetic protein-2 expression [16]. Diabetes accelerates bone loss via suppression of bone formation. STZ-treated mice show decreased expression of osteoblast-specific genes (runt-related transcription factor 2, TGF-β) compared with non-diabetic mice [27]. Sclerostin is a WNT antagonist secreted by osteocytes that binds to LDL receptor-related protein 5/6 and inhibits osteoblast differentiation [28]. STZ-treated mice showed increased sclerostin levels in serum and matrix embedded cells of the callus after femur fracture [29]. In the present study, the DP group also showed elevated proportions of sclerostin-positive osteocytes compared with the C group. Interestingly, the proportion of sclerostin-positive osteocytes in the DPS group was lower than the DP group, suggesting that SIM may improve bone formation in type 1 diabetes by inhibiting sclerostin expression in osteocytes.

Our previous work showed severe alveolar bone loss and inhibition of alveolar bone formation with elevated sclerostin expression on day 20 in rats with STZ-induced diabetes and periodontitis [23]. Therefore, to monitor the effect of systemic SIM treatment during the time of these changes, the timepoints selected for this study were days 3, 10, and 20. However, we did not observe beneficial effects of SIM treatment on alveolar bone resorption and formation in type 1 diabetic rats with periodontitis. This suggests that systemic SIM administration during 20 days may not have beneficial effects on the severe alveolar bone loss in type 1 diabetes with periodontitis. The effects of systemic SIM treatment have evaluated under various conditions. For example, systemic SIM treatment for 8 weeks reduced the number of osteoclasts in rats with LPS-induced periodontitis [5]. Further, systemic SIM treatment for 4 weeks decreased RANKL and colony stimulating factor 2 expressions in periodontal tissues of obese rats with LPS-induced periodontitis, suggesting an inhibitory effect of SIM on expression of osteoclastogenesis factors in rats with metabolic syndrome [14]. Finally, systemic SIM administration for 2 months prevented alveolar bone loss in ovariectomized rats with periodontitis [11]. The lack of response to systemic SIM treatment in terms of alveolar bone loss in type 1 diabetic rats with periodontitis in the present study may be due to differences in SIM administration time or the systemic disease model accompanied with periodontitis. To clarify the effect of SIM, it will be necessary to study prolonged systemic SIM administration.

## Conclusions

In rats with type 1 diabetes and periodontitis, we did not observe significant prevention of periodontitis-induced alveolar bone loss during 20 days of systemic SIM administration. However, SIM attenuated tibial bone loss at early stage with weak bone loss, but not at late stage with sever bone loss. Moreover, SIM decreased the number of osteoclasts and proportion of sclerostin-positive osteocytes in tibiae. Together, our results suggest that the effect of SIM on tibial bone loss may be related to the inhibition of osteoclast formation and sclerostin expression in type 1 diabetic rats with periodontitis. Our results provide useful information for establishing SIM as a therapeutic strategy for systemic diseases with severe alveolar bone loss.

## Abbreviations

SIM: simvastatin
LPS: lipopolysaccharide
BMD: bone mineral density
STZ: streptozotocin
C: control
DP: diabetes with periodontitis
DPS: diabetes with periodontitis treated with simvastatin
TG: triglyceride
CHO: cholesterol
LDL: low-density lipoprotein
EDTA: ethylenediaminetetraacetic acid
H&E: hematoxylin and eosin
CEJ: cementoenamel junction
ABC: alveolar bone crest
ROI: region of interest
m-CT: micro-computed tomography
BVF: bone volume fraction
BSD: bone surface density
Tb. N: trabecular number
TRAP: tartrate-resistant acid phosphatase
RANKL: receptor activator of nuclear factor kappa-B ligand
TGF: transforming growth factor
